# Preoperative treatment with levosimendan helps to evaluate myocardial reserves in cardiosurgical patients with chronic heart failure

**DOI:** 10.1186/cc14230

**Published:** 2015-03-16

**Authors:** A Eremenko, M Babaev, S Fedulova, S Dzemeshkevish

**Affiliations:** 1 FBSI 'Petrovsky NRCS', Moscow, Russia

## Introduction

The aim of the study was to assess the possibility of preoperative levosimendan (LS) administration in myocardial reserve evaluation and choice of the method of surgical treatment in patients with chronic heart failure (CHF).

## Methods

LS was used in 107 (female and male) patients (mean age 53 ± 3 years) as a component of CHF therapy to prepare them for the surgical intervention (2 to 4 days before surgery). In total, 44.9% of the patients had CHF caused by noncoronarogenic dilated cardiomyopathy and 55.1% by ischemic cardiomyopathy. Indication for LS therapy was left ventricular ejection fraction (EF) <35% (28 ± 6%). Seventy percent of patients had mitral insufficiency (MI), grades II to IV, 63% tricuspid insufficiency (TrI), grades II to III, 84% pulmonary hypertension (PH), 47% arterial hypertension, grades II to III, and 27% of the patients had left ventricular aneurysm. Mean level of BNP was 1,803 ± 124 pg/ml. LS was administered as i.v. infusion in doses of 0.025 to 0.1 μg/kg/minute without previous bolus injection. Mean duration of infusion was 27.5 ± 5.3 hours. After infusion all patients underwent control assessment of values. All patients were operated: 25 (23.3%) underwent reverse cardiac remodeling, 63 (58.9%) myocardium revascularization (MR) with mitral or aortic valve replacement, 17 (15.9%) MR and/or resection of left ventricular aneurism and two (1.9%) heart transplantation.

## Results

Heterogeneity of LS effects was registered in a number of values. The most significant positive effect which allowed one to evaluate myocardial reserve was demonstrated by decrease of PPA (93.5% of patients) and increase of EF (77.6% of patients). The most significant changes were also noted in decrease of TrI, PH and MI (in 53.2%, 36.6% and 36% of patients, respectively). In 69.2% of patients with noncoronarogenic dilated cardiomyopathy the effect of LS exposure was marked. In the majority of patients with ischemic cardiomyopathy the effect was moderate. In case of the absence of LS-positive effect, perioperative use of mechanical circulatory support was considered.

## Conclusion

Preoperative use of LS allows one to evaluate myocardial reserves and prepare high-risk patients with CHF for surgery. Our findings may serve as one of the additional criteria to choose the type of surgical treatment: reconstructive surgery (with or without perioperative mechanical circulatory support) or heart transplantation.

